# Silencing RPL11 attenuates acute kidney injury by suppressing tubular apoptosis and macrophage-driven inflammation

**DOI:** 10.3389/fimmu.2025.1642446

**Published:** 2025-08-15

**Authors:** Qian Dong, Huan Xu, Pengjie Xu, Jiang Liu, Zhouji Shen, Yabin Li

**Affiliations:** ^1^ Department of Nephrology, The Affiliated Lihuili Hospital of Ningbo University, Ningbo, China; ^2^ School of Life Sciences and Technology, Tongji University, Shanghai, China

**Keywords:** RPL11, acute kidney injury (AKI), tubular dysfunction, nanoparticle targeting, immune dysregulation

## Abstract

**Background:**

Acute kidney injury (AKI) remains a life-threatening syndrome with elusive molecular drivers. Although ribosomal proteins such as RPL11 are increasingly recognized for their extra-ribosomal functions, their roles in AKI pathogenesis remain unexplored.

**Methods:**

The comprehensive multi-omics analysis of mouse AKI kidneys combined scRNA-seq and RNA-seq to identify core regulatory factors. Based on cisplatin induced AKI, a HK-2 cell model was established by siRNA transfection silencing RPL11, while *in vivo* kidney targeted silencing was achieved using LyP-1 modified nanoparticles encapsulating si-RPL11. Technologies such as Western blotting, qPCR, and IVS fluorescence imaging ensure the successful construction of cell and animal models. Functional testing includes CCK-8, EdU assay, flow cytometry, TUNEL assay, qPCR, ELISA, and histopathological techniques to evaluate cell proliferation, apoptosis, and inflammatory cytokine levels.

**Results:**

RPL11 was identified as the core gene with AKI-specific upregulation in proximal tubules. RPL11 expression correlated with AKI severity and showed positive associations with Scr/KIM-1. The specific silencing of RPL11 in HK-2 cells was successfully induced and the LYS-1 peptide-modified cationic liposome nanoparticles were stable in quality and could target the renal tissue of AKI mice to silence RPL11. The experimental results have jointly confirmed that RPL11 suppressed proliferation, accelerated apoptosis, amplified inflammation and aggravated tubular necrosis and CD68 macrophage infiltration *in vitro* and *in vivo*.

**Conclusion:**

RPL11 drives AKI progression by orchestrating tubular dysfunction, apoptosis, and immune dysregulation. Our renal-targeted nano-intervention validates RPL11 as a therapeutically actionable target, providing a novel strategy for biomarker-guided AKI management.

## Introduction

Acute kidney injury (AKI) is characterized by a rapid kidney dysfunction, leading to dysregulation of nitrogenous waste excretion, electrolyte imbalances, acid-base disturbances, and fluid retention. This intricate clinical construct harbors diverse etiologies, including but not limited to infection, ischemia, obstruction, and nephrotoxic drugs (1, 2). According to the current popular definitions, AKI refers to a rapid increase of creatinine and a rapid decrease in urine output within days (3). Globally, AKI contributes to 1.7 million deaths, affecting approximately 13.3 million people annually. It complicates 10–15% of hospital admissions and exceeds 50% in intensive care settings (4). Major etiologies include nephrotoxic agents (e.g., cisplatin chemotherapy) and ischemia-reperfusion injury (IRI) (5). Despite its clinical significance, no targeted pharmacotherapies exist beyond supportive care, such as vasoactive agents and renal replacement therapy. This highlights an urgent need for mechanism-based interventions (6, 7). Therefore, we should take timely intervention measures to prevent the condition from deteriorating to an irreversible damage stage.

AKI frequently occurs secondary to severe trauma, burns, or major surgery. In these conditions, impaired wound healing and infections arising during prolonged recovery contribute to AKI development and increase its incidence (8, 9). A systematic review and meta-analysis of trauma patients admitted to the ICU confirmed that abdominal injury and shock are risk factors for AKI, with 24% of ICU-admitted trauma patients developing AKI (10, 11). In addition, studies have demonstrated that even minor injuries confer high AKI risk in elderly patients (12). Interestingly, recent research has implicated ribosomal proteins in wound pathology. Ribosomal protein L11 (RPL11) functions as a critical ribosomal stress sensor and translocates to the nucleolus to modulate key pathways governing cell survival, proliferation, and inflammation (13). Single-cell RNA sequencing of wound exudate has revealed upregulated RPL11 expression in non-healing wounds, where it disrupts protein synthesis and impedes tissue repair (14). This suggested its potential as a universal biomarker for pathological non-healing wounds. Furthermore, as the first identified ribosomal protein that stabilizes and activates tumor protein 53 (p53), RPL11 is recognized as a non-redundant nodal regulator of the tumor-suppressive axis (15, 16). During ribosomal stress, it stabilizes p53 primarily by inhibiting murine double minute 2 (MDM2)-mediated degradation, thereby preserving basal p53 activity for tumor surveillance and genomic maintenance (17, 18). Additionally, RPL11 suppresses tumor growth by inhibiting c-Myc translation (19, 20). To date, although no studies have directly investigated RPL11 in AKI, its demonstrated pharmacological activities—including modulation of cell proliferation, apoptosis, inflammation, and immune responses in injury and oncology and Diamond-Blackfan anemia—provide crucial theoretical support for exploring its potential protective mechanisms in AKI.

In the treatment of renal disease, gastrointestinal disorders, and cancer, nanotechnology has demonstrated a higher degree of efficacy compared to small-molecule medicines (21, 22). For instance, the ROS-responsive nanoparticle NPS_BG_@Cur with high ROS sensitivity and bioavailability, was engineered to deliver the autophagy activator curcumin (Cur) specifically to injured kidneys. This approach established autophagy-driven lipid metabolism correction as a viable therapeutic strategy (23). In addition, polyphenol-based nano-formulations mitigate AKI through coordinated reno-protective effects, including ameliorating tubular damage, restoring glomerular filtration, and enhancing tissue regeneration (24). Emerging evidence suggests that rationally engineered nano-delivery systems could enhance therapeutic precision in AKI by improving drug bioavailability and targeting renal repair pathways, although clinical translation requires further validation.

To clarify the pathological role and regulatory mechanism of RPL11 in AKI, we plan to carry out cross-level validation studies, including clinical sample analysis alongside experiments *in vitro* and *in vivo*. In addition, we combine multi-omics screening, gain/loss of function experiments and kidney-targeted nano-therapy platform. This study attempts to reveal the roles of RPL11 in AKI and elucidate the potential association between ribosomal stress response and AKI progression. These initial results provide preliminary mechanistic foundations for future RPL11-targeted renal protective strategies and lay important groundwork for advancing mechanistic understanding in critical nephrology.

## Materials and methods

### Single-cell RNA sequencing analysis

The scRNA-seq dataset (GSE139506) was acquired from Gene Expression Omnibus (GEO) database, comprising kidney cells from AKI and control mice. The raw data processing was conducted using Seurat package (v4.0) in R. Cells were quality-filtered to exclude those with <200 or >6,000 detected genes or >15% mitochondrial gene content. Data normalization and dimensionality reduction were carried out by means of SCTransform and principal component analysis (PCA) in sequence. Cell clusters were identified using shared nearest neighbors (SNN) modular optimization (resolution=0.5). Proximal tubule cells were annotated based on canonical marker genes (Kap, Lrp2, Slc27a2, Slc34a1) using a method known as uniform manifold approximation and projection (UMAP). The genes demonstrating significant differential expression between AKI and control groups were computed using the Wilcoxon rank-sum test, with significant differentially expressed genes (DEGs, |log2Fold Change (FC)| >1 and *p <*0.05, Benjamini-Hochberg).

### Transcriptome sequencing

Transcriptomic data (GSE202753) were downloaded from GEO, including 4 AKI and 3 control mouse kidney samples. The raw reads were aligned to the Mus musculus reference genome (GRCm38) using STAR (v2.7.10a), and gene counts were quantified via featureCounts. A differential expression analysis was conducted with the use of R statistical software and the DESeq2 (v1.30.1) package., applying independent filtering and Wald testing. DEGs were selected under stringent thresholds: |log2FC| >2 and *p <*0.05. PCA and sample correlation heatmaps were generated to evaluate batch effects and inter-group consistency. For functional enrichment, a Gene Set Enrichment Analysis (GSEA) was conducted on the Hallmark gene set of the Molecular Characteristics Database (MSigDB), and the pathways were ranked by normalized enrichment score (NES) and false discovery rate (FDR <0.05).

### Clinical samples characteristics and processing

Renal tissue, blood, and urine samples were collected from patients diagnosed with AKI, and the normal renal tissues (nephrectomy specimens from non-diseased regions) were utilized in the control experiments. These collected tissues were used for immunohistochemistry (IHC) and immunoblotting. Blood and urine samples were centrifuged (3,000 ×g, 15 min) to isolate serum and supernatant, respectively.

The prepared sections (4 μm) underwent antigen retrieval and then blocking. The samples were incubated with a primary antibody (1:250, Abcam) against RPL11 at 4°C for 12-16 h and. Following this initial incubation, the samples were transferred to a secondary antibody (1:500, Dako) that was HRP-conjugated. The secondary antibody was allowed to incubate with the samples for 1 h. Diaminobenzidine (DAB) was used for chromogenic detection, and nuclei were counterstained with hematoxylin. The frozen tissues were then subjected to homogenization using RIPA buffer, with the addition of protease inhibitors. The protein lysates underwent separation via SDS-PAGE, followed by transfer onto PVDF membranes, and probed with anti-RPL11 (1:1200, Cell Signaling Technology, CST) and β-actin (1:4000, CST).

### Cell culture

Human kidney 2 (HK-2) cells were cultivated in the medium (DMEM/F12 + 10% fetal bovine serum (FBS) + 1% penicillin/streptomycin), at 37°C and 5% CO_2._ For siRNA transfection, the HK-2 cells were seeded in 6-well plates (3 × 10^5^ cells per well) and transfected at 60–70% confluency using Lipofectamine 3000 reagent. RPL11-specific siRNA (20 nM, sequence validated by BLAST and synthesized by a commercial vendor) and non-targeting siRNA (si-NC) were diluted in Opti-MEM and incubated with Lipofectamine 3000 (1:1 ratio, 15 min, room temperature (RT)). The cells were transfected for 6 h, followed by replacement with fresh complete medium. After 48 h, Western blotting (WB) and qPCR were employed to confirm the transfection efficiency, revealing a >70% knockdown of RPL11 protein and mRNA compared to si-NC (normalized to β-actin).

For AKI modeling, transfected cells were exposed to cisplatin (20 μM) for 24 h. The treat group (si-RPL11 + AKI + rh-RPL11) received recombinant human RPL11 protein (50 ng/mL, optimized concentration) 2 h prior to cisplatin exposure. All experimental groups included triplicate biological replicates, and cells were maintained under standardized conditions (passage number ≤15, mycoplasma-free confirmed by PCR).

### Cell viability measurement

The cell viability was detected using CCK-8 assay. The HK-2 cells were seeded into 96-well plates (8000 cells per well). The cells were divided into four groups: CTRL (untreated), si-NC + AKI (transfected with negative control siRNA followed by cisplatin-induced AKI at 20 μM for 24 h), si-RPL11 + AKI (transfected with RPL11 siRNA plus cisplatin), and si-RPL11 + AKI + rh-RPL11 (RPL11 siRNA, cisplatin, and recombinant human RPL11 protein). Following a 24-hour treatment period, the CCK-8 operating fluid was added to each well (100 μL, 1-2 h).

### EdU assay

The initial step was to seed the HK-2 cells in 24-well plates (5×10^4^ cells per well) EdU solution (10 μM final concentration) was incubated with cells for 2.5 h. Then, the HK-2 cells were fixed and permeabilized using 4% paraformaldehyde (15 min) and 0.3% Triton X-100 (10 min) in sequence. Then, the fixed and permeabilized cells were incubated with a Click reaction cocktail (containing Alexa Fluor 594) for 0.5 h in the dark. The nuclei were subjected to staining with DAPI (1 μg/mL, 10 min), and imaged using a fluorescence microscope. The intensity of the red fluorescence (EdU-positive) and the blue fluorescence (total nuclei) was quantified using ImageJ. The proliferation rates are expressed as the percentage of EdU-positive cells. All steps were performed in triplicate.

### Flow cytometry

The HK-2 cells from all groups were collected by trypsinization (without EDTA). The cells were washed 3 times with PBS, and subsequently resuspended in binding buffer (100 μL). Finally, Annexin V-FITC (5 μL) and propidium iodide (PI, 5 μL) were used for staining HK-2 cells. These samples were analyzed within 1 h using a flow cytometer.

### Enzyme-linked immunosorbent assay

Cell culture supernatants from all experimental groups (CTRL, si-NC + AKI, si-RPL11 + AKI, si-RPL11 + AKI + rh-RPL11) were collected and centrifuged (300 × g, 10 min, 4°C). Commercial human ELISA kits (e.g., R&D Systems or Abcam) were purchased to quantify the concentration of IL-1β and IL-18.

### AKI mouse model

SPF-grade C57BL/6J mice (male, 8 weeks) were purchased from Vital River (Beijing). The Animal Care and Use Committee of Shanghai Tenth People’s Hospital formally approved all animal experimental protocols. The mice were randomly assigned to 5 groups after acclimation. (*n* = 6): WT, WT+AKI, si-RPL11, si-RPL11+AKI, and si-RPL11+AKI+rhRPL11 group. WT group: Wild-type mice without treatment. WT + AKI group: WT mice injected intraperitoneally (i.p.) with cisplatin (20 mg/kg, single dose); si-RPL11 group: WT mice receiving RPL11 siRNA-loaded LNPs (1 mg/kg, i.v., every 3 days for 1 week); si-RPL11 + AKI group: RPL11 siRNA-treated mice injected with cisplatin (20 mg/kg, i.p.); si-RPL11 + AKI + rhRPL11 group: RPL11 siRNA + cisplatin-treated mice supplemented with recombinant human RPL11 protein (5 mg/kg, i.p., daily for 3 days post-cisplatin). Cisplatin (1 mg/mL) was dissolved in sterile 0.9% saline and administered intraperitoneally at 20 mg/kg. The mice in the control group received equal-volume saline.

The total duration of the experiment was 7 days, during which the mice could freely obtain water and food. At the start of the experiment, the WT and WT+AKI groups received an intraperitoneal (i.p.) injection of si-NC LNPs. The remaining three groups (si-RPL11, si-RPL11+AKI, and si-RPL11+AKI+rhRPL11) received an i.p. injection of an equivalent volume of si-RPL11 LNPs. Three days later, the WT +AKI, si-RPL11+AKI, and si-RPL11+AKI+rhRPL11 groups were administered with cisplatin (20 mg/kg, i.p.) to induce the AKI model. Three days after cisplatin administration, the mice were anesthetized, and a comprehensive set of samples was collected, including blood and various tissues.

### Lipid nanoparticle preparation

Cationic LNPs were formulated using a solvent injection method. Briefly, a lipid mixture of DOTAP, DOPE, and Cholesterol (molar ratio optimized to 4:3:3 based on preliminary screening) was dissolved in ethanol. siRNA (1 mg/mL in RNase-free buffer, targeting RPL11 or non-targeting control) was diluted in citrate buffer (pH 4.0). The lipid solution was rapidly injected into the aqueous siRNA phase under vigorous vortexing (1:3 v/v lipid-to-siRNA ratio) and incubated (0.5 h, 37°C) to facilitate self-assembly. For renal-targeted delivery, pre-formed LNPs were modified using a commercial kidney-targeting platform (e.g., RiboBio Kidney-targeting siRNA Delivery Kit) by incubating with 1% (w/w) PEGylated renal-targeting ligands (e.g., kidney-specific aptamers) for 1 h at 25°C.

### Characterization methods and quality control

Size and Zeta Potential: LNPs were diluted in PBS (1:100) and analyzed by dynamic light scattering (DLS, Malvern Zetasizer). Only batches with an average hydrodynamic diameter at 80–120 nm and polydispersity index (PDI) <0.2, and zeta potential at +15 to +30 mV were selected.

Encapsulation Efficiency (EE%): Unencapsulated siRNA was removed via centrifugal filtration (100 kDa cutoff, 12,000 ×g, 30 min). siRNA concentration was quantified fluorometrically using the RiboGreen assay (Thermo Fisher). Batches with EE% >80% were selected.

Serum Stability: LNPs were subjected to an incubation process in PBS with 10% FBS (37°C for 30, 60, 90, and 120 min), respectively. The samples were electrophoresed on a 2% agarose gel (100 V, 30 min) to assess siRNA integrity. Stable LNPs showed minimal free siRNA leakage.

### Histopathologic analysis

Kidney tissues underwent fixation in 10% formalin and subsequent embedding in paraffin. To observe the pathological alterations of the renal tissue, hematoxylin and eosin (H&E) staining was employed.

### Quantitative real-time polymerase chain reaction

Total RNA was isolated from HK-2 cells and renal cortical tissues using TRIzol reagent. The reverse transcription process was conducted with 1 μg of RNA, employing the Prime Script RT Reagent Kit with gDNA Eraser (Takara) in accordance with the stipulated conditions: 42°C for 2 min, 37°C for 15 min, and 85°C for 5 s. qPCR reactions were conducted in triplicate using SYBR Green Master Mix (Roche) on a CFX96 Real-Time PCR System (Bio-Rad) according to the following protocol: 95°C for 30 s, 40 cycles of 95°C for 5 s and 60°C for 30 s, followed by a melt curve analysis (65–95°C, 0.5°C increments).

### Immunohistochemistry

The prepared kidney sections were incubated with a primary antibody against RPL11 (PA5-101381, Thermo) and a secondary antibody in sequence. The images were scanned, and IHC scores were quantified using ImageJ. The calculation of the score is determined by the following formula: score = (number of pixels in a zone) * (score of the zone)/total number of pixels in the image.

### Tunnel assay

Initially, the paraffin-embedded renal tissue sections underwent dewaxing, followed by thorough rehydration. The deparaffinized sections suffered proteinase K treatment, dUTP labeling and DAPI staining. The images were obtained using a scanner (3DHISTECH). The fluorescence intensity was quantified using ImageJ.

### Immunofluorescence staining

The sections were cooled to RT after antigen repair. and then incubated with 5% donkey serum (1 h, RT). These blocked sections were incubated with the primary antibodies CD68 (rabbit polyclonal, 1:150, CST) at 4°C for 12-16 h. Secondary Antibodies: Washed slides were treated with species-specific Alexa Fluor-conjugated antibodies: Donkey anti-mouse IgG-Alexa Fluor 488 (1:500, for RPL11) and Donkey anti-rabbit IgG-Alexa Fluor 594 (1:500, for CD68). Then, incubation for 1 h at RT, protected from light. Sections were stained with DAPI (1 μg/mL, 5 min), washed with PBS. Fluorescence images were captured using a confocal microscope (Zeiss LSM 880) with consistent settings (20x objective, 1024×1024 resolution). Z-stack imaging (5 slices/section) ensured signal accuracy.

### Data statistics

All the data was expressed as the mean ± standard deviation (M ± SD) and underwent processing and visualization using GraphPad Prism 7. Further statistical analyses were performed via one-way ANOVA and *P <*0.05 was regarded as having significant differences.

## Results

### scRNA-seq and RNA-seq in renal tissues of mice with AKI

**Figure 1:**
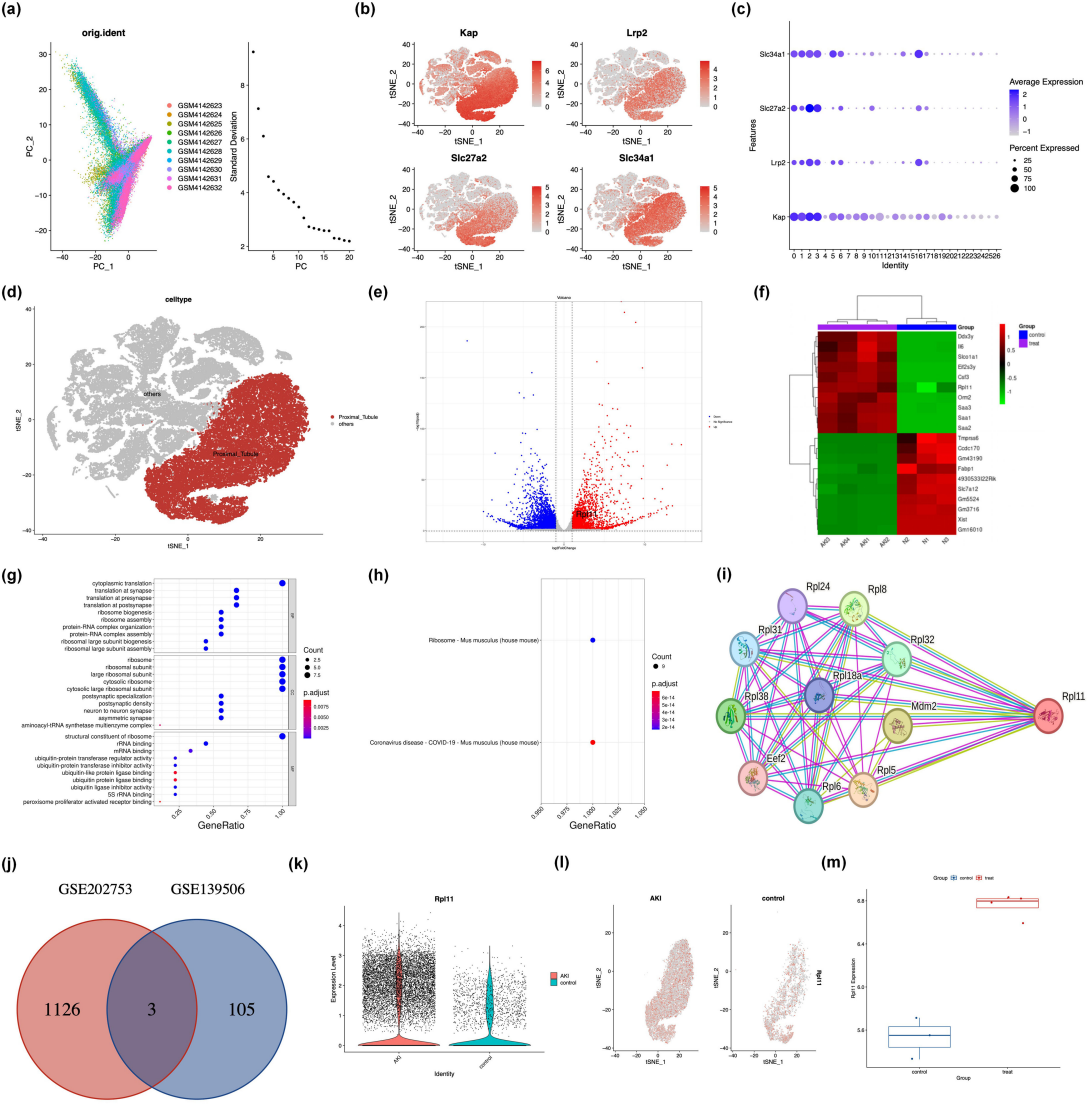
Integrated analysis of single-cell and transcriptome sequencing in murine acute kidney injury. **(a)** Principal component analysis (PCA) of scRNA-seq data from AKI kidney samples; **(b)** Spatial distribution of canonical cell markers within the renal cellular atlas; **(c)** Quantitative assessment of marker gene localization in kidney compartments; **(d)** Identification and annotation of proximal tubule epithelial cells; **(e)** Volcano plot of DEGs from RNA-seq; **(f)** Heatmap of DEGs from RNA-seq; **(g)** Bar graph of GO functional enrichment analysis of DEGs; **(h)** Scatter plot of GO term enrichment; **(i)** PPI network of ribosome-associated DEGs; **(j)** Venn diagram intersecting DEGs from scRNA-seq and RNA-seq; **(k)** The expression density of the RPL11 gene in PTC; **(l)** The tSNE plot of the RPL11 gene; **(m)** The mRNA expression of the RPL11 (*P <0.05, **P <0.01, ***P <0.001, ****P <0.0001).

This study employed integrated scRNA-seq and RNA-Seq to comprehensively characterize the molecular features of kidney tissue in a murine model of AKI. Through scRNA-seq analysis of kidney samples from AKI mice and Control group mice, we systematically mapped the renal cell atlas. PCA dimensionality reduction visualization (**Figure 1a**) clearly demonstrated significant differences in cellular composition between the Control and AKI groups, with clustering distribution exhibiting a high degree of concordance with intercellular correlation. Based on the expression patterns of known renal proximal tubular cell (PTC)-specific markers (Kap, Lrp2, Slc27a2, Slc34a1) (**Figures 1b–d**), we successfully identified and isolated the target PTC subpopulation. Within this target subpopulation, applying the screening criteria of |log2FC >1| and *p <*0.05, we identified a total of 108 significantly DEGs.

RNA-Seq analysis similarly revealed extensive gene expression differences between renal tissues of Control and AKI group mice. A volcano plot (**Figure 1e**) intuitively displayed the differential expression, identifying 1129 significant DEGs (*p <*0.05), among which 716 genes were upregulated and 513 genes were downregulated following AKI induction. Functional enrichment analysis indicated that these DEGs were significantly enriched for genes associated with carcinogenesis and the hematopoietic system, such as RPL11 and Tmprss6 (**Figure 1f**). Further GO annotation analysis demonstrated that in terms of BP and CC, the DEGs were predominantly enriched in ribosome-related pathways; while at the MF level, they were significantly enriched for ubiquitin-protein ligase activity-related functions (**Figure 1g**). To further investigate the biological pathways involved by the DEGs, we performed GSEA. The top significantly enriched pathways identified by GSEA were constructed into an interaction network (**Figures 1h, i**). Notably, network topology analysis revealed that RPL11 occupied a key hub position within the network, suggesting its potential role as a core regulator in the AKI pathological process.

Integrating findings from the single-cell resolution and whole-tissue level, we took the intersection of the 108 DEGs identified within the target PTC subpopulation by scRNA-seq and the 1332 DEGs obtained from RNA-seq. The analysis result showed that only three genes, B2m, Ugt2b37, and Rpl11, exhibited upregulated expression (**Figure 1j**). Based on an in-depth investigation of related biological functions using the NCBI database, we focused on the ribosome-associated gene Rpl11 as the core subject for subsequent research. Further exploration of the scRNA-seq data confirmed that RPL11 protein expression was significantly elevated within PTCs of the AKI group (**Figures 1k, l**). This finding was strongly supported at the whole-tissue level by RNA-seq data (**Figure 1m**).

Collectively, these integrated analyses of scRNA-seq and RNA-seq highlights the significant potential of RPL11 as a novel diagnostic or prognostic biomarker for AKI.

### Validation of RPL11 involvement in AKI using clinical samples

**Figure 2:**
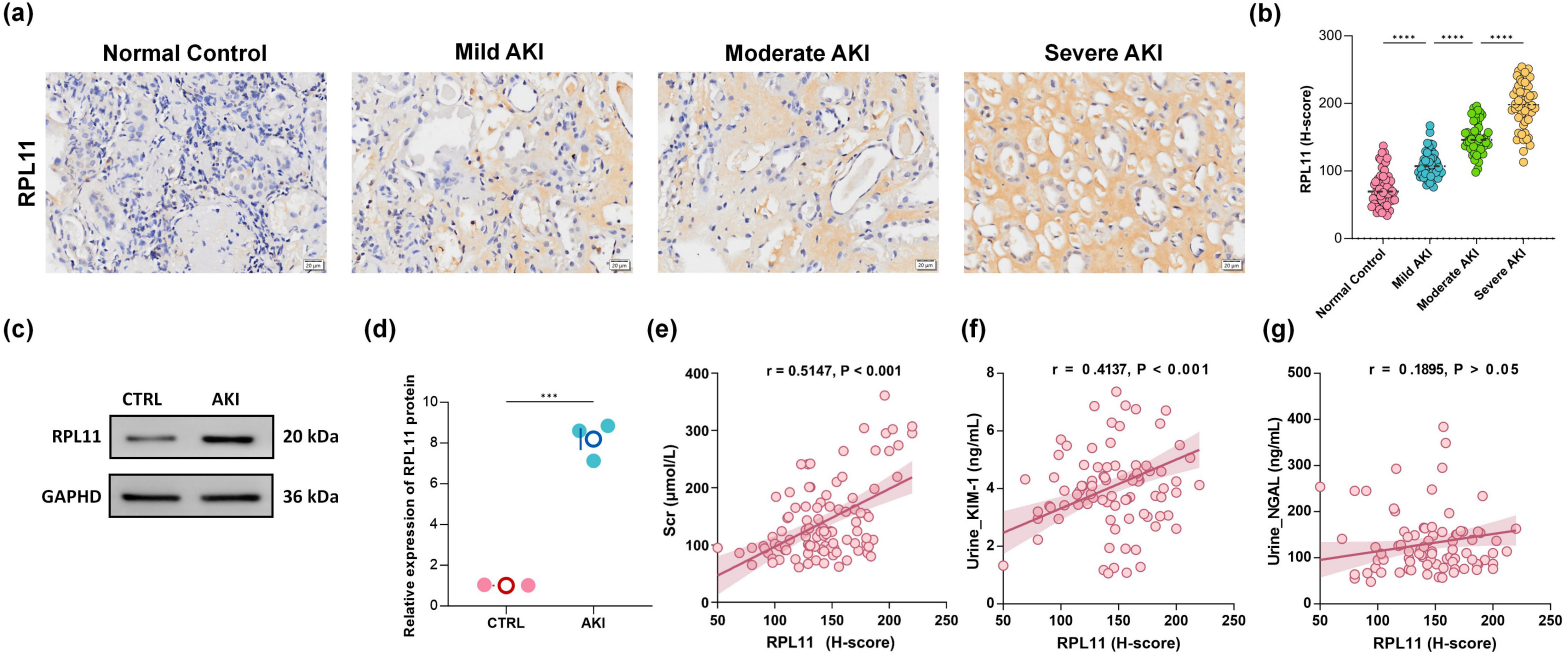
The expression of RPL11 in clinical kidney samples. **(a)** Representative immunohistochemical images of RPL11 in kidneys of different AKI stages; **(b)** Quantitative results of H-core immunohistochemistry for RPL11; **(c)** Western blotting protein band of RPL11 in the kidneys; **(d)** Quantitative results of RPL11 protein by Western blotting; Correlation analysis of **(e)** Scr, **(f)** urine KIM-1, and **(g)** urine NGAL with the expression level of RPL11 protein (***P <0.001, ****P <0.0001).

To validate the multi-omics findings that implicate RPL11 is involved in AKI, we investigated RPL11 expression in renal tissue from clinical AKI patients. IHC was performed to localize and quantify RPL11 expression across different stages of human AKI. Compared to normal renal tissue, RPL11 expression intensity was significantly enhanced in AKI kidneys (**Figures 2a, b**). Critically, this upregulation exhibited a pronounced positive correlation with the severity of renal injury. Consistent with the IHC staining, WB confirmed a significant elevation of RPL11 protein levels within AKI patient kidneys (**Figure 2c**). Semi-quantitative densitometry revealed statistically significant differences (**Figure 2d**). These findings, aligning with the integrated transcriptomic and single-cell sequencing analyses, collectively demonstrate that RPL11 is abnormally expressed in the kidneys of AKI patients. To elucidate the potential pathological consequences of RPL11 dysregulation in AKI, we analyzed the correlation between RPL11 expression levels (quantified by IHC H-score) and established clinical biomarkers of AKI. These biomarkers included serum creatinine (Scr), urinary neutrophil gelatinase-associated lipocalin (NGAL) and kidney injury molecule-1 (KIM-1). Correlation analysis revealed distinct patterns: While RPL11 H-scores showed no significant correlation with urinary NGAL, they demonstrated moderate positive correlations with both Scr and urinary KIM-1 levels (**Figures 2e–g**). These results observed aberrantly high RPL11 expression in AKI, which is consistent with the experimental findings from scRNA-Seq and RNA-Seq.

### Silencing RPL11 in renal tubular epithelial cells can alleviate cisplatin-induced inhibition of renal tubular proliferation and inflammatory injury

**Figure 3:**
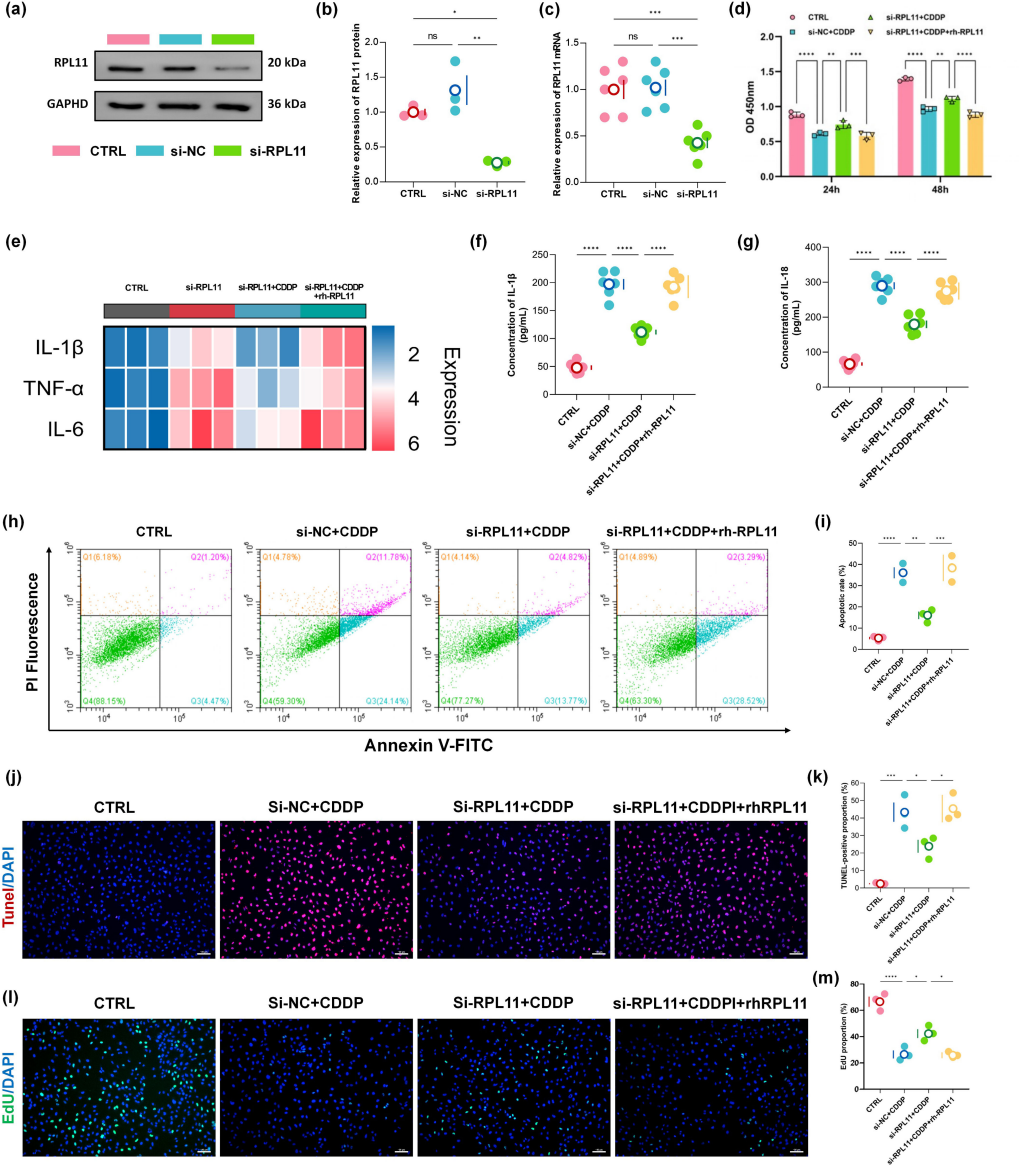
Functional impact of RPL11 on HK-2 cells. **(a)** Representative Western blot of RPL11 protein expression following si-RPL11 transfection; **(b)** Quantitative densitometric analysis of RPL11 protein levels; **(c)** qPCR analysis of RPL11 mRNA expression post-transfection; **(d)** Cell viability assessed by CCK-8 assay under indicated treatments; **(e)** Representative images of EdU staining; **(f)** Quantitative analysis of EdUχ cell proportions; **(g)** Flow cytometry plots of apoptosis using Annexin V/PI dual staining; **(h)** Quantification of total apoptotic rates; **(i)** TUNEL staining; **(j)** Semi-quantitative analysis of TUNEL fluorescence intensity; **(k)** Heatmap of pro-inflammatory cytokine mRNA expression in HK-2 cells; ELISA determination of **(l)** IL-1β content and **(m)** IL-18 in HK-2 cells (*P <0.05, **P <0.01, ***P <0.001, ****P <0.0001).

To elucidate the pathological consequences of RPL11 expression in renal tubular epithelial cells, we established RPL11-knockdown models by transfecting HK-2 cells with specific siRNA *in vitro*. No significant difference in RPL11 expression was observed between the si-NC and CTRL groups using WB. In contrast, RPL11 protein was markedly reduced in the si-RPL11 group, with densitometric quantification confirming significant downregulation (*P <*0.05, **Figures 3a, b**). Concurrently, qPCR demonstrated a synchronous decrease in RPL11 mRNA transcription, collectively validating the successful establishment of the RPL11-inhibition model (*P <*0.001, **Figure 3c**).

Cellular proliferation dynamics were assessed using CCK-8 and EdU assays. Cisplatin (CDDP) treatment for 24 h and 48 h significantly suppressed HK-2 cell proliferation (*P <*0.0001, **Figure 3d**). Notably, RPL11 silencing partially reversed CDDP-induced proliferative inhibition, whereas exogenous recombinant RPL11 protein (rh-RPL11) reinstated the suppression of cell viability (*P <*0.01, *P <*0.001 or *P <*0.0001, **Figure 3d**). EdU assays corroborated these findings: the si-RPL11 + CDDP group exhibited a significantly higher proportion of DNA replication-positive cells than the si-NC + CDDP group, while the rh-RPL11 group reproduced proliferative arrest (*P <*0.05, **Figures 3l, m**). These results indicate an inverse correlation between RPL11 expression levels and proliferative capacity. Apoptosis analysis revealed that CDDP robustly induced late-stage apoptosis in HK-2 cells (apoptotic rate: 33.31% *vs* CTRL group 5.53%, *P <*0.0001, **Figures 3h, i**). RPL11 silencing significantly reduced the total apoptotic rate (*P <*0.01, **Figures 3h, i**), whereas rh-RPL11 treatment promoted early apoptotic progression (increased Annexin Vχ/PI⁻ cell population). TUNEL assays further substantiated this trend: the si-RPL11 group displayed significantly attenuated DNA fragmentation fluorescence intensity compared to the CDDP group, while the rh-RPL11 group restored high-intensity apoptotic signals (*P <*0.05, *P <*0.05, **Figures 3j, k**). These data demonstrate that RPL11 exacerbates tubular cell injury by activating apoptotic pathways. To further define RPL11’s role in cellular injury, inflammatory cytokine transcription was evaluated by qPCR. Heatmap analysis demonstrated that CDDP dramatically upregulated pro-inflammatory genes (IL-1β, TNF-α and IL-6). RPL11 silencing suppressed this hyperactivation, and rh-RPL11 partially restored their elevated expression (**Figure 3e**). ELISA of cell supernatants revealed that secretion levels of IL-1β and IL-18 mirrored mRNA expression patterns (**Figures 3f, g**), confirming that RPL11 amplifies inflammation at both transcriptional and translational levels, thereby aggravating renal injury.

In conclusion, employing a bidirectional regulatory strategy (siRNA-mediated silencing and recombinant protein rescue), we demonstrate that RPL11 inhibits the cell proliferation of renal tubular cells, induces their apoptosis, and promotes the transcription and release of inflammatory factors, exacerbating the deterioration of the inflammatory microenvironment and accelerating AKI pathogenesis.

### Validation of RPL11 in AKI *in vivo*

**Figure 4:**
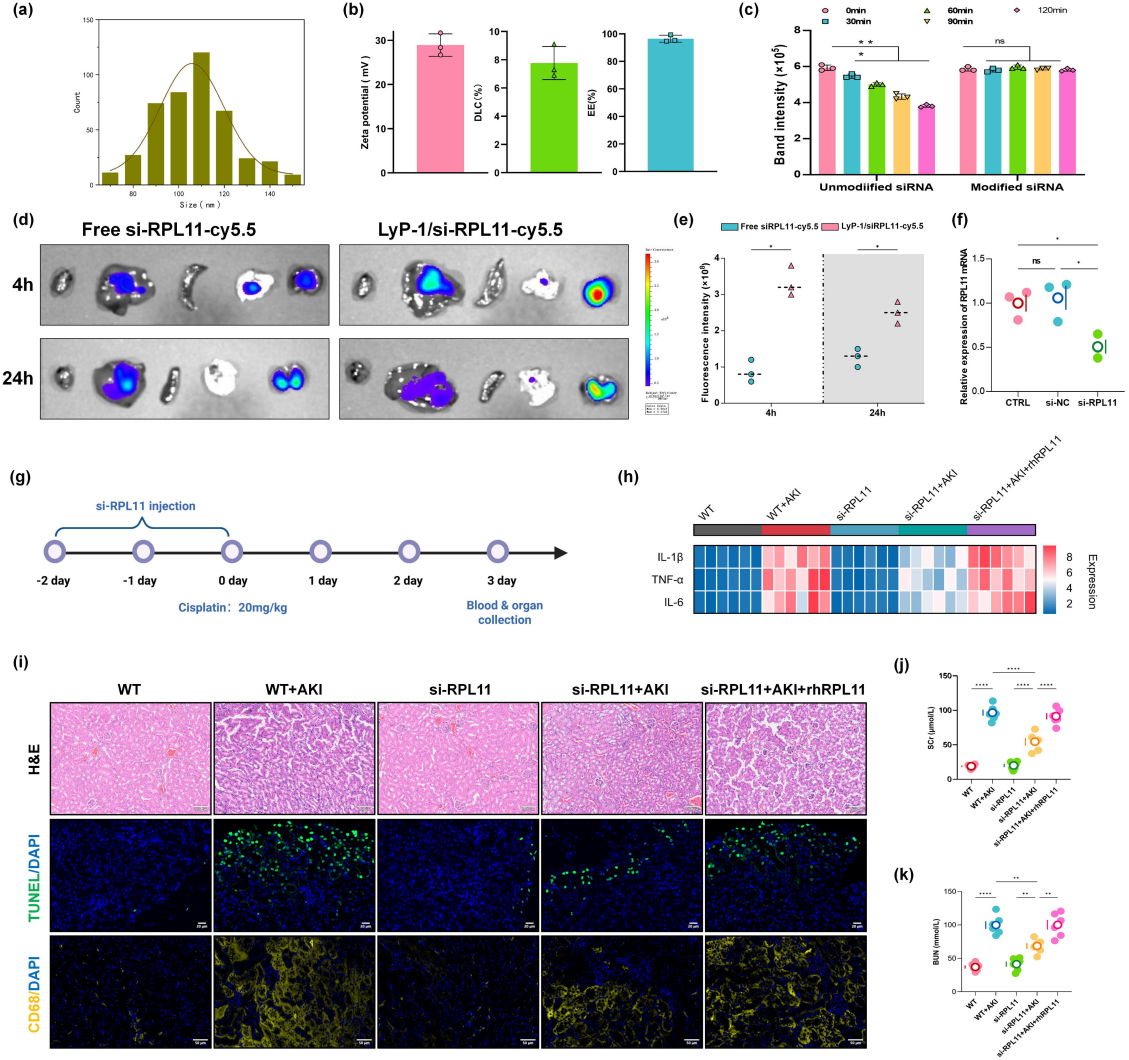
Validation of RPL11 in wild-type mice with AKI *in vivo*. **(a)** Size distribution of LNPs; **(b)** Zeta potential, Drug loading efficiency and encapsulation efficiency; **(c)** siRNA release rate; **(d)** Representative photos of the distribution of Cy5-labeled si-RPL11 in various tissues; **(e)** Quantitative results of fluorescence imaging; **(f)** qPCR detection of RPL11 expression knockdown efficiency in renal tissue; **(g)** Experimental drug administration scheme for mice; **(h)** Heatmap of pro-inflammatory cytokine mRNA expression in renal tissues; **(i)** Representative images of HE staining, TUNEL fluorescence staining and immunofluorescence; **(j)** Measurement of Scr in mice; **(k)** Measurement of BUN in mice (ns, non-significant; *P <0.05, **P <0.01, ****P <0.0001).

To elucidate the effects of RPL11 in AKI pathogenesis, we established a kidney-targeted RPL11-knockdown mouse model using LyP-1 peptide-modified cationic lipid nanoparticles (LNPs) encapsulating si-RPL11. This strategy leveraged the specific binding affinity of LyP-1 to renal proximal tubules to enhance renal targeting efficiency. Nanoparticle characterization revealed the size distribution is 80–120 nm (PDI <0.2) and the zeta potential are +15 mV to +30 mV with the characteristic of cationic carriers (**Figures 4a, b**). Unlike the unmodified group, no significant difference in release kinetics within 120 min in the Modified group (*P >*0.05), which confirms formulation robustness (**Figure 4c**).

To assess *in vivo* the renal targeting efficiency and gene silencing validation of the LNPs, Cy5-labeled si-RPL11 was administered via tail vein injection. Representative images and IVIS imaging collectively demonstrated significantly higher fluorescence intensity in kidneys than in liver, lung, spleen, or heart at 24 h (**Figure 4d**). Semi-quantitative analysis further revealed a time-dependent increase in renal accumulation (**Figure 4e**). qPCR confirmed efficient RPL11 mRNA knockdown (*P <*0.05) in renal tissue, validating the targeting system (**Figure 4f**).

Wild-type mice received intraperitoneal injections of si-NC or si-RPL11 LNPs for 3 consecutive days, followed by a single cisplatin dose (20 mg/kg) to induce AKI (**Figure 4g**). These are the key findings as follows. Compared with si-RPL11+AKI group, si-RPL11 reduced Scr and BUN by 48.2% and 37.6%, respectively, indicating restored glomerular filtration and renal function (*P <*0.0001 or *P <*0.01, **Figures 4j, k**). The mRNA levels of IL-1β, TNF-α and IL-6, were significantly suppressed (**Figure 4h**), which is consistent with *in vitro* data. Observing the pathological sections of the kidney tissue, H&E staining revealed attenuated tubular injury in si-RPL11 mice, characterized by preserved epithelial cell alignment, reduced vacuolar degeneration and granular casts, and minimal tubular necrosis. In contrast, WT+AKI controls exhibited severe epithelial disorganization and widespread necrosis (**Figure 4i**). TUNEL intensity decreased markedly in si-RPL11 mice *vs*. si-RPL11+AKI mice, while rh-RPL11 rescue restored high apoptosis signals (**Figure 4i**). CD68 serves as a specific surface marker of macrophages, facilitating interactions between macrophages and pathogens, as well as with other immune cells (25, 26). Elevated expression of CD68 specifically indicates the occurrence of an inflammatory response. Therefore, immunofluorescence was utilized to investigate the effects of RPL11 deletion on the infiltration of immune cells. The results demonstrated that the expression of CD68 in WT + AKI group underwent a substantial enhancement, while it was superseding si-RPL11 group, and rh-RPL11 partially reversed the reduction effect of immune cell infiltration induced by si-RPL11 (**Figure 4i**). This indicates that RPL11 can regulate immune cells in the kidneys and intervene in the immune response of the body.

Collectively, our *in vivo* evidence establishes RPL11 as a pathogenic driver within renal cells, where its activity induces tubular epithelial apoptosis, recruits pro-inflammatory macrophages, and disrupts the local immune microenvironment, which provides a compelling rationale for the therapeutic targeting of si-RPL11 in AKI.

## Discussion

AKI represents a critical clinical syndrome associated with high morbidity and mortality. However, its underlying molecular drivers remain incompletely characterized. This study systematically elucidates the core role of RPL11 in AKI pathogenesis through the integration of multi-omics analyses. Initially, by integrating single-cell sequencing and transcriptomic profiling, RPL11 was identified as a significantly upregulated intersecting gene within renal tubular cells of AKI mice. IHC and WB analyses confirmed that RPL11 expression intensity positively correlates with AKI clinical stage and is significantly associated with established AKI biomarkers, including Scr and KIM-1. This association suggests that RPL11 dysregulation may influence key AKI progression pathways, potentially impacting inflammation, cellular stress responses, or proliferation. Thus, aberrantly high RPL11 expression emerges as both a potential diagnostic biomarker and a modulator of disease progression. It will be a therapeutic target with significant promise for intervention.

In cisplatin-induced HK-2 cell models, RPL11 knockout reversed cisplatin-mediated inhibition of cell proliferation, as demonstrated by CCK-8 and EdU assays. Flow cytometry and TUNEL assays further indicated that RPL11 depletion significantly reduced apoptosis and suppressed pro-inflammatory cytokine secretion. Conversely, recombinant RPL11 administration exacerbated these cellular injury phenotypes. In an *in vivo* AKI murine model, targeted delivery of si-RPL11 via LyP-1 peptide-modified nanoparticles significantly attenuated renal dysfunction. This improvement was evidenced by marked reductions in Scr and BUN levels, decreased renal infiltration of CD68^+^ macrophages, and diminished renal tissue inflammation and cellular necrosis. These results suggest that RPL11 silencing breaks the tubule-macrophage positive feedback loop, a core pathogenic axis in AKI. Collectively, these findings demonstrate that RPL11 drives AKI progression by orchestrating the dysregulation of tubular cell proliferation, apoptosis, inflammatory responses, and immune cell activation. And they provide novel mechanistic insights into AKI pathogenesis and highlight RPL11-targeted therapeutic strategies as promising avenues for intervention.

NGAL serves as a gold standard biomarker for early AKI diagnosis, detecting renal tubular damage within hours of injury. However, our analysis revealed no significant correlation between RPL11 H-scores and urinary NGAL levels (*r* = 0.1895, *P*> 0.05). This discrepancy may stem from fundamental differences in their temporal kinetics and etiological bias. First, NGAL peaks within 2-4 hours post-injury, whereas KIM-1 remains elevated for 24-48 hours post-injury. The significant correlation between RPL11 and KIM-1 (*r* = 0.4137, *P <*0.001) corroborates that RPL11 requires >24 hours to accumulate to detectable levels. This temporal disparity in biomarker response likely underlies the lack of correlation observed between NGAL and RPL11. Furthermore, while NGAL demonstrates excellent sensitivity for early AKI detection, its expression exhibits significant variation across AKI subtypes. For example, NGAL levels >104 μg/ml are strongly associated with intrinsic renal AKI, whereas levels <47 μg/ml make intrinsic renal AKI unlikely (27). In contrast, RPL11 demonstrates constitutively high expression across diverse etiologies, encompassing pre-renal, intrinsic renal, and post-renal AKI. Increasing the clinical sample size is crucial for a more comprehensive assessment of the correlation between NAGL and RPL11.

Studies indicate that ribosomal proteins undergo significant alterations during injury repair processes. Within 18 hours following sciatic nerve injury, ribosomal proteins accumulate markedly in axons. It suggests their potential direct involvement in localized translational reprogramming during the regenerative phase (28). Furthermore, RPL11 exhibits sustained upregulation in multiple non-healing wound models, including those driven by aging (98-week-old mice), diabetes (db/db mice), and inflammation (LPS-induced wounds) (14). This dysregulation may impede tissue repair by suppressing ribosome biogenesis. Clinical epidemiological investigations further reveal that patients undergoing major surgeries face substantially elevated risks of secondary AKI (8, 29, 30). Among them, abdominal surgery patients are particularly vulnerable (95% CI: 10.9–16.4%) (29). The core pathological mechanism involves impaired wound healing and infection. Given RPL11’s demonstrated function in coordinating the balance between tissue regeneration and apoptosis within non-malignant pathological contexts, systematically exploring its role in AKI is needed. It may not only uncover novel pathological mechanisms, but also potentially offer new theoretical foundations for ribosome stress-targeted renal protection.

Consistent with the established anti-proliferative and pro-apoptotic roles in cancer, the study demonstrates that RPL11 is pathologically upregulated in renal tubular cells during AKI, and it acts as a central driver of injury progression. We found that RPL11 depletion via targeted siRNA delivery significantly reversed the proliferation inhibition and apoptosis of tubular cells. And it attenuated the secretion of pro-inflammatory cytokines *in vitro* and *in vivo*. Furthermore, targeted knockdown of RPL11 using LyP-1-modified nanoparticles markedly improved renal function, diminished CD68 macrophage infiltration, and reduced renal inflammation and necrosis. These findings bridge the critical gap in understanding the role of RPL11 beyond oncology. Moreover, they robustly establish its mechanistic contribution to AKI pathogenesis.

Although this study confirms that the functions mediated by RPL11 in AKI may be closely associated with p53, the precise underlying mechanisms require further elucidation. Subsequent research should focus on dynamically monitoring RPL11-p53 axis activity across different stages of AKI progression. Their temporal correlation with renal function markers, such as Scr and urinary NGAL, also needed to be analyse. Furthermore, studies should utilize renal tubular cell-specific RPL11 knockout models to determine whether RPL11 regulation of p53 directly governs tubular cell apoptosis. Finally, administer p53-specific agonists or antagonists to comprehensively assess the regulatory role in renal tissue repair capacity and the inflammatory microenvironment. These in-depth mechanistic investigations will provide critical evidence to confirm the central role of the RPL11-p53 pathway in influencing AKI progression. They will further strengthen the rationale for targeting RPL11 therapeutically and laying the foundation for developing pathway-specific therapeutics.

Current therapeutic strategies for AKI, including renal replacement therapy (RRT), pharmacologic interventions, and biologic agents, face significant limitations compromising efficacy (31). RRT modalities rapidly clear metabolic waste but exhibit poor macromolecular toxin clearance, device dependency, and high mortality in low-resource settings (32). Pharmacologic approaches carry substantial risks. Early bicarbonate administration increases mortality by 76%, while diuretics merely augment urine output without functional recovery (33). Novel biologics like anti-myoglobin antibodies or NLRP3-inhibiting peptides suffer from poor renal targeting and instability (34). To overcome these barriers, we developed an LNP system that synergizes targeted delivery with multifactorial pathophysiology modulation. Our LNP design features are as followed. The traditional cationic lipid core could facilitate the release of siRNA within lysosomes, thereby forming a stable complex with good biocompatibility and low toxicity (35). LyP-1 peptide conjugation enables p32/gC1qR receptor-mediated uptake, which is upregulated on injured tubular epithelia. Particle parameters were optimized to 80 nm diameter and cationic zeta potential (15 to 30 mV) to evade pulmonary entrapment and prolong circulation half-life (36). In cisplatin-induced AKI models, LyP-1-LNP/si-RPL11 elicited superior renoprotection: Scr and BUN decreased by 48.2% and 37.6%, respectively, and CD68^+^ macrophage infiltration reduced. Mechanistically, it concurrently suppressed tubular apoptosis, inflammasome activation, and immune recruitment, breaking the apoptosis-inflammation vicious cycle. This formulation provides precise nephroprotection via a tripartite mechanism. With its superior renal targeting and multi-pathway therapeutic effects, it represents a promising drug candidate for AKI.

A significant limitation is kidney-targeted RPL11 knockdown via systemic nanoparticle delivery rather than conditional tubular epithelial-specific knockout models. Although siRNA achieved high renal efficiency, two constraints persist. On the one hand, incomplete organ specificity permits low-efficient silencing effects in extrarenal organs (e.g., heart and liver). And potential off-target effects may indirectly affect renal phenotype through metabolic disorders. On the other hand, insufficient cellular resolution within kidney——contributions from glomerular/interstitial cells still cannot be excluded, though renal tubular cells account for more than 80% of the parenchyma. Thus, observed kidney injury may partially originate from impaired glomerular filtration or inflammatory crosstalk. Furthermore, constrained by the experimental timeline and sample size, AKI evaluation in this study relied primarily on biomarker evaluation rather than histological scoring. Follow-up studies will incorporate standardized histological analysis of renal biopsy specimens to enhance diagnostic accuracy comprehensively. Additionally, RPL11 silencing improved function but lacked quantification of p53 and downstream targets at the gene and protein level. Consequently, reno-protection cannot be definitively attributed to p53 modulation versus off-target effects. Althought substantial literature supports the RPL11-p53 interaction, future studies employing genetically modified cell or animal models are necessary to assess the functions of the association. Thereby a more comprehensive and in-depth elucidation of the underlying mechanism could be provided. Finally, the unresolved long-term safety profile of cationic LNPs is another key limitation. Potential lipid accumulation, chronic inflammatory responses, and degradation product toxicity require extended *in vivo* studies (37, 38).

## Conclusion

By integrating multi-omics, clinical samples, cellular and animal approaches, we identify RPL11 as a multi-functional effector of AKI pathogenesis. It could impair tubular proliferation, accelerate apoptosis and inflammation, and recruit injury-amplifying macrophages. The kidney-targeted siRNA delivery system validates RPL11’s therapeutic potential, providing a blueprint for ribosomal protein-targeted interventions in inflammatory diseases. In summary, our findings demonstrate that si-RPL11 confers reno-protection by promoting tubular proliferation, suppressing inflammatory responses, and enhancing immune defense mechanisms.

## Data Availability

The original contributions presented in the study are included in the article/**Supplementary Material**. Further inquiries can be directed to the corresponding author.
