# Supplementary material for: Silencing RPL11 attenuates acute kidney injury by suppressing tubular apoptosis and macrophage-driven inflammation
Source: Front Immunol. 2025 Aug 15;16:1642446. doi: 10.3389/fimmu.2025.1642446 (PMC12394147; doi:10.3389/fimmu.2025.1642446)
Supplement: Supplementary file 1 [file DataSheet1.docx]

Supplementary Figure. 1 Effects of rh-RPL11 on cell viability of si-RPL11+AKI HK-20 cells.

**Additional Information of Clinical Samples Characteristics.**

This study enrolled a total of 100 participants (51 males, 49 females) at the Affiliated Lihuili Hospital of Ningbo University between 2022 and 2023. Among them, 30 healthy subjects were assigned to the control group, and the remaining 70 patients with AKI were included in the AKI group. Renal tissue, venous blood, and midstream urine samples were collected from all AKI patients within 24 hours of diagnosis. The median age of the participants was 53 years (range: 24-82 years). Etiologies of AKI included pre-renal (*n* = 26), renal (*n* = 26), and post-renal (*n* = 18). Detailed information can be found in **Supplementary Table 1**.All patients had baseline normal renal function (serum creatinine <106 μmol/L) with no prior history of renal replacement therapy or immunosuppressant use. The study was approved by the Ethics Committee (Approval No.: KY2024SL295-01), and written informed consent was obtained from all participants.

**Supplementary Table 1. Baseline Characteristics of the AKI Clinical Cohort**

| **Patient** | **Sex** | **Age** | **Group** | **Etiology** | **Patient** | **Sex** | **Age** | **Group** | **Etiology** |
| --- | --- | --- | --- | --- | --- | --- | --- | --- | --- |
| P001 | M | 59 | control | normal | P027 | F | 62 | AKI | pre-renal |
| P002 | M | 76 | control | normal | P028 | F | 63 | control | normal |
| P003 | M | 43 | AKI | renal | P029 | F | 57 | AKI | renal |
| P004 | M | 82 | AKI | pre-renal | P030 | F | 50 | AKI | pre-renal |
| P005 | F | 71 | AKI | pre-renal | P031 | M | 58 | control | normal |
| P006 | F | 67 | AKI | renal | P032 | M | 31 | AKI | renal |
| P007 | M | 49 | control | normal | P033 | F | 53 | AKI | renal |
| P008 | F | 81 | AKI | renal | P034 | F | 68 | AKI | pre-renal |
| P009 | F | 57 | AKI | renal | P035 | F | 71 | AKI | pre-renal |
| P010 | F | 58 | AKI | post-renal | P036 | M | 56 | AKI | post-renal |
| P011 | M | 53 | control | normal | P037 | M | 38 | control | normal |
| P012 | M | 58 | AKI | renal | P038 | F | 73 | control | normal |
| P013 | M | 67 | AKI | renal | P039 | M | 63 | control | normal |
| P014 | M | 54 | AKI | renal | P040 | M | 35 | control | normal |
| P015 | F | 57 | AKI | renal | P041 | F | 47 | AKI | renal |
| P016 | M | 43 | AKI | pre-renal | P042 | F | 37 | AKI | post-renal |
| P017 | M | 40 | AKI | post-renal | P043 | M | 70 | AKI | post-renal |
| P018 | M | 55 | AKI | pre-renal | P044 | F | 32 | control | normal |
| P019 | F | 64 | control | normal | P045 | F | 45 | AKI | pre-renal |
| P020 | M | 43 | control | normal | P046 | F | 31 | AKI | pre-renal |
| P021 | M | 55 | control | normal | P047 | F | 61 | AKI | post-renal |
| P022 | M | 46 | AKI | renal | P048 | M | 57 | control | normal |
| P023 | M | 68 | AKI | renal | P049 | F | 67 | control | normal |
| P024 | F | 60 | AKI | renal | P050 | M | 69 | control | normal |
| P025 | F | 71 | AKI | post-renal | P051 | F | 44 | control | normal |
| P026 | M | 24 | AKI | pre-renal | P052 | M | 52 | AKI | post-renal |

**Supplementary Table 1 Continued.**

| **Patient** | **Sex** | **Age** | **Group** | **Etiology** | **Patient** | **Sex** | **Age** | **Group** | **Etiology** |
| --- | --- | --- | --- | --- | --- | --- | --- | --- | --- |
| P053 | M | 41 | control | normal | P077 | M | 62 | control | normal |
| P054 | M | 51 | control | normal | P078 | F | 42 | AKI | renal |
| P055 | F | 76 | control | normal | P079 | F | 47 | AKI | renal |
| P056 | F | 53 | AKI | pre-renal | P080 | F | 47 | AKI | post-renal |
| P057 | F | 33 | AKI | pre-renal | P081 | M | 37 | AKI | pre-renal |
| P058 | F | 60 | AKI | post-renal | P082 | M | 43 | AKI | post-renal |
| P059 | M | 30 | control | normal | P083 | M | 37 | control | normal |
| P060 | F | 48 | AKI | pre-renal | P084 | F | 34 | AKI | renal |
| P061 | M | 72 | AKI | pre-renal | P085 | M | 47 | AKI | pre-renal |
| P062 | F | 74 | AKI | pre-renal | P086 | M | 37 | control | normal |
| P063 | M | 41 | AKI | renal | P087 | M | 36 | AKI | renal |
| P064 | M | 30 | AKI | post-renal | P088 | F | 30 | AKI | post-renal |
| P065 | F | 34 | AKI | post-renal | P089 | M | 60 | AKI | pre-renal |
| P066 | F | 38 | control | normal | P090 | M | 46 | AKI | pre-renal |
| P067 | M | 41 | AKI | pre-renal | P091 | F | 59 | AKI | renal |
| P068 | F | 62 | control | normal | P092 | F | 51 | AKI | post-renal |
| P069 | M | 67 | control | normal | P093 | M | 53 | AKI | pre-renal |
| P070 | M | 43 | AKI | pre-renal | P094 | F | 65 | control | normal |
| P071 | F | 75 | AKI | renal | P095 | M | 63 | AKI | renal |
| P072 | F | 67 | AKI | renal | P096 | F | 52 | AKI | renal |
| P073 | F | 56 | AKI | post-renal | P097 | M | 57 | AKI | post-renal |
| P074 | F | 56 | AKI | post-renal | P098 | F | 52 | AKI | pre-renal |
| P075 | M | 42 | AKI | renal | P099 | M | 26 | AKI | pre-renal |
| P076 | F | 47 | AKI | pre-renal | P100 | M | 62 | control | normal |
